# Prediction of Outcome From Adult Bacterial Meningitis in a High-HIV-Seroprevalence, Resource-Poor Setting Using the Malawi Adult Meningitis Score (MAMS)

**DOI:** 10.1093/cid/ciw779

**Published:** 2016-12-02

**Authors:** Emma C. Wall, Mavuto Mukaka, Matthew Scarborough, Katherine M. A. Ajdukiewicz, Katharine E. Cartwright, Mulinda Nyirenda, Brigitte Denis, Theresa J. Allain, Brian Faragher, David G. Lalloo, Robert S. Heyderman

**Affiliations:** 1 Malawi-Liverpool-Wellcome Trust Clinical Research Programme, College of Medicine, University of Malawi, Blantyre; 2 Liverpool School of Tropical Medicine; 3 Division of Infection and Immunity, University College London, United Kingdom; 4 Mahidol-Oxford Clinical Research Unit, Faculty of Tropical Medicine, Mahidol University, Bangkok, Thailand; 5 Oxford Centre for Tropical Medicine and Global Health, Nuffield Department of Medicine Research Building, University of Oxford; 6 Oxford University Hospitals; 7 University of Manchester Academic Health Science Centre, North Manchester General Hospital; 8 Sheffield Teaching Hospitals, United Kingdom; 9 Department of Emergency Medicine, Queen Elizabeth Central Hospital; 10 Department of Medicine, College of Medicine, University of Malawi, Blantyre, Malawi

**Keywords:** bacterial meningitis, HIV, Africa, outcome, severity score

## Abstract

**Background.:**

Acute bacterial meningitis (ABM) in adults residing in resource-poor countries is associated with mortality rates >50%. To improve outcome, interventional trials and standardized clinical algorithms are urgently required. To optimize these processes, we developed and validated an outcome prediction tool to identify ABM patients at greatest risk of death.

**Methods.:**

We derived a nomogram using mortality predictors derived from a logistic regression model of a discovery database of adult Malawian patients with ABM (n = 523 [65%] cerebrospinal fluid [CSF] culture positive). We validated the nomogram internally using a bootstrap procedure and subsequently used the nomogram scores to further interpret the effects of adjunctive dexamethasone and glycerol using clinical trial data from Malawi.

**Results.:**

ABM mortality at 6-week follow-up was 54%. Five of 15 variables tested were strongly associated with poor outcome (CSF culture positivity, CSF white blood cell count, hemoglobin, Glasgow Coma Scale, and pulse rate), and were used in the derivation of the Malawi Adult Meningitis Score (MAMS) nomogram. The C-index (area under the curve) was 0.76 (95% confidence interval, .71–.80) and calibration was good (Hosmer-Lemeshow C-statistic = 5.48, *df* = 8, *P* = .705). Harmful effects of adjunctive glycerol were observed in groups with relatively low predicted risk of poor outcome (25%–50% risk): Case Fatality Rate of 21% in the placebo group and 52% in the glycerol group (*P* < .001). This effect was not seen with adjunctive dexamethasone.

**Conclusions.:**

MAMS provides a novel tool for predicting prognosis and improving interpretation of ABM clinical trials by risk stratification in resource-poor settings. Whether MAMS can be applied to non-HIV-endemic countries requires further evaluation.

## Summary

The Malawi Adult Meningitis Score prediction tool accurately estimates risk of clinical outcome from bacterial meningitis in sub-Saharan Africa. Clinical trial analysis by risk stratification reveals more severe outcomes in low-risk groups receiving adjunctive glycerol compared to placebo.

**(See the Editorial Commentary by Quagliarello on pages 420–1.)**

Acute bacterial meningitis (ABM) in sub-Saharan Africa is common and associated with high rates of death and comorbidity in both adults and children [1, 2]. Throughout the African continent, bacterial meningitis is predominately caused by *Streptococcus pneumoniae* [3–5], with the exception of the “meningitis belt” Sahel region, where seasonal epidemic meningococcal meningitis also occurs [6]. In Malawi, the incidence of ABM in adults is 12 per 100000, and the associated mortality is 54% compared with 20%–30% in Europe and the United States [1, 2, 7, 8]. Despite intensive efforts, high mortality remains unchanged region-wide; adjunctive treatments such as corticosteroids and glycerol are ineffective and possibly harmful [9–12].

To improve ABM outcome, interventional trials testing new treatment approaches and resource-appropriate standardized clinical algorithms are urgently required. A robust outcome prediction tool would help optimize these processes and provide a basis to examine why previous adjunctive interventions have been unsuccessful [13].

Well-validated scores used for risk stratification in life-threatening infection include Confusion Urea Respiratory Rate age 65 (CURB-65) for pneumonia, and Acute Physiology And Chronic Health Evaluation (APACHE II) and Sepsis-related Organ Failure Assessment (SOFA) for sepsis [14–17]. Two good-quality scores have been reported for ABM in developed settings [18, 19]. A meningitis score has been developed in the African subcontinent [20] but was developed specifically for outcome prediction during meningococcal outbreaks.

We have previously identified clinical predictors of death from adult ABM in Malawi [1]. In the present study, we grouped these outcome predictors to generate and validate a severity score nomogram for use in resource-poor settings. We used the validated score to analyze data from large placebo-controlled trials of adjunctive dexamethasone and glycerol in Malawi, to better understand why these treatments were ineffective.

## PATIENTS AND METHODS

### Patient Selection

**Figure 1.**
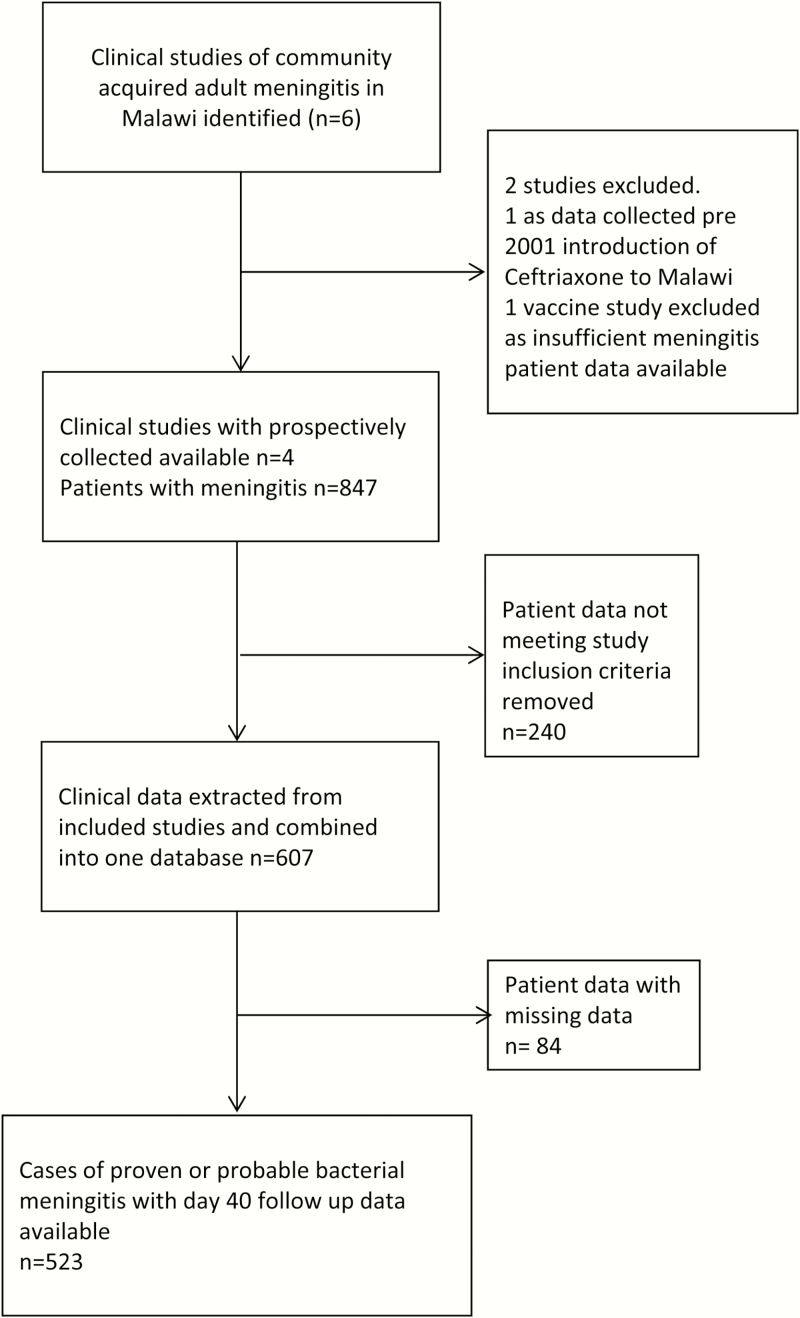
Consolidated Standards of Reporting Trials (CONSORT) diagram demonstrating selection of data for the Malawi Meningitis Database.

Clinical data from the Malawi Meningitis Database (MMD) [1], and patient data from a recent clinical trial of goal-directed therapy (ISRCTN96218197) were used (Figure 1). All patients had been enrolled in clinical studies of adult ABM in Queen Elizabeth Central Hospital in Blantyre, Malawi, between 2000 and 2013 (Figure 1). The inclusion criteria for the MMD were age >14 years with proven cerebrospinal fluid (CSF) infection on culture, polymerase chain reaction, or Gram stain of bacteria known to cause meningitis (proven meningitis), or appropriate clinical history <5 days with a CSF white blood cell (WBC) count >50 cells/µL and >50% neutrophils (probable meningitis). In human immunodeficiency virus (HIV)–coinfected individuals, the CSF WBC count threshold for probable meningitis was lowered to >5 cells/µL, (100% neutrophils), to include HIV-coinfected individuals with low CD4 counts who mount a weak CSF inflammatory response in ABM [1, 4].

Cases were excluded if there was CSF evidence of *Cryptococcus neoformans* or *Mycobacterium tuberculosis*, CSF WBC count >50% lymphocytes with no documented prehospital antibiotics, or trial-specific exclusion criteria such as diabetes for the glycerol trial [9, 10]. Diagnostic testing for viral meningitis was not available and is an uncommon cause of neurological infection in our center [21]. MMD patients randomized to receive glycerol were excluded from the nomogram development, as glycerol receipt was an independent predictor of poor outcome [10].

### Ascertainment of Clinical Characteristics and Outcomes

All prospective clinical trial data were utilized with permission from the principal investigator. Individual study protocols were examined; only identical variables across the studies were collected (data collected in the same units, at similar time points, using the same normal ranges). All studies were undertaken in the same hospital, using the same laboratories and similar case record forms. Physiological data are from the first recording to minimize both heterogeneity and biases associated with delays to antibiotic therapy, and increase applicability for clinical use in the region. Outcome was measured as death or survival at day 40 postadmission to account for additional acute postdischarge mortality, which has been reported to occur due to late complications in 5%–10% of initial survivors of ABM [9, 22]. Data on in-hospital delays and morbidity (principally deafness) were collected variably across the studies and were not included to minimize heterogeneity.

### Ethical Approvals

All contributing clinical studies were granted ethical approval by the College of Medicine Research and Ethics Committee, University of Malawi, and the Liverpool School of Tropical Medicine Research Ethics Committee and conformed to institutional guidelines. All trial participants or named patient guardians provided written informed consent.

### Selection of Predictor Variables

A literature search was performed to determine predictor variables for adults with bacterial meningitis [1]. Individual predictors of mortality from the published analysis of the MMD (coma, seizures, anemia, and tachycardia) [1] and variables associated in other studies with poor outcome (age, sex, HIV status, CSF WBC count, and CSF culture) were finally used [7, 19, 23, 24].

### Statistical Methods, Derivation of the Nomogram, and Validation Methods

Data were summarized as follows: continuous normally distributed data using means and standard deviations, skewed data using medians and interquartile range, and categorical data using percentages. Analyses using a univariate logistic regression model were performed on all relevant covariates and factors to assess any association with day 40 outcome. The unadjusted estimates were reported using odds ratios (ORs) and 95% confidence intervals (CIs). Multivariable analyses followed on the discovery cohort using a logistic regression model to assess factors and covariates independently associated with the day 40 outcome; this model included all variables with statistical significance on univariate analysis. Variables with high proportions (>50%) of missing data were excluded to reduce risks of overfitting. All analyses were performed using IBM SPSS software version 20.0 (IBM Statistics, Armonk, New York) and Stata software version 13.0 (StataCorp, College Station, Texas). Statistical significance was set at *P* < .05.

All variables with significance in the multivariable model were used to model the predictive tool, using R software version R-i386 3.0.3 (www.r-project.org) using *rms* package and the *lrm* and *nomogram* commands.

The developed model was validated to estimate the potential model performance. Because external data for validation have not yet been identified, an internal validation procedure was performed using bootstrapping (5000 samples), based on calibration, and discrimination [25, 26]. This procedure was selected to optimize the number of cases available while minimizing risks of overfitting.

A logistic regression model was used to obtain the predicted deaths. The Hosmer-Lemeshow C-statistic was used to assess calibration; low insignificant Hosmer-Lemeshow C-statistic indicated good fit [27]. The calibration assessment was supplemented by the use of a Brier score (range 0 and 1); an α value <.05 (5% significance level) was used to determine model fit [28, 29]. A receiver operating characteristic (ROC) curve was plotted and used to calculate the validated concordance index (C-index). The ROC curve was obtained with the C-index (area under the curve [AUC]) and associated 95% CIs. The sensitivity and specificity of the model is based on a cutoff of 50% risk score. The individual Malawi Adult Meningitis Score (MAMS) was calculated for patients using IBM SPSS software version 20.0.

### Analysis of Clinical Trial Data Using MAMS

The MAMS was calculated for all patients who had been recruited to clinical trials testing adjunctive dexamethasone and glycerol. Cases were risk stratified by quartile risk of poor outcome, and separated by placebo or intervention. Case fatality rate (CFRs) were calculated within each quartile group and compared to the predicted risk of poor outcome; statistical significance was determined using χ^2^ test. Data were analyzed using both original complete case and bootstrapped data.

## RESULTS

### Clinical Characteristics of the Patients

Data on patients meeting the inclusion criteria were available for 607 ABM cases and were used for univariate analyses. Five hundred ninety-three patients had CSF results available; of these, 384 (65%) were CSF culture positive (Supplementary Table 1). Complete case data were available for 523 cases of bacterial meningitis, which were used for the multivariable analyses and development of the nomogram. Mean age was 32.5 years (SD, 10.8 years), 50.7% (308/607) were female, 87% (506/584) were HIV coinfected, and 31% (171/551) had an acute seizure within the first 6 hours of admission. Presenting characteristics by outcome group are detailed in Table 1.

**Table 1.** Results of the Univariate Analysis and Predictors of Outcome From the Complete Case Data

Parameter	Day 40		Univariate Analysis (Unadjusted)		Multivariable Analysis (Adjusted)^a^	
	Alive	Dead	Odds Ratio (95% CI)	*P* Value	Odds Ratio (95% CI)	*P* Value
Sample size n = 607	273	320				
Age, y, mean (SD)	31.5 (10.9)	32 (10.6)	1.01 (1.00–1.03)	.01	1.00 (.95–1.06)	.84
>40 y	42 (14.6)	53 (16.6)	1.30 (.67–2.5)	.42		
Sex, male	136 (47)	163 (50.9)	1.17 (.85–1.62)	.32		
HIV status						
Infected	226 (78)	280 (87)	0.79 (.32–1.95)	.61		
Unknown	10 (3.5)	13 (4.1)				
Admission out of hours	122 (42.5)	139 (43.9)	1.20 (.68–1.28)	.40		
Unknown	30 (10.5)	29 (9.1)				
GCS, mean (SD)	12 (2.8)	9.9 (3.5)	0.82 (.78–.86)	<.001	0.77 (.72–.83)	<.001
Acute seizure episodes						
1 seizure	45 (16)	70 (22)	0.61 (.30–1.23)	.16		
2 seizures	18 (6.3)	37 (11.6)	0.75 (.33–1.70)	.49		
Temperature, °C, mean (SD)	38.2 (1.1)	38.4 (1.3)	1.17 (1.02–1.34)^b^	.02	^c^	
SpO_2_, %, mean (SD)	95.5 (2.5)	96 (6.5)	0.90 (.82–1.00)^b^	.07	^c^	
Pulse rate, beats/min, mean (SD)	98.6 (19)	104.8 (19.8)	1.01 (1.00–1.02)^b^	<.001	1.01 (1.00–1.03)	.01
MAP, mm Hg, mean (SD)	89.9 (15.1)	90.5 (16.7)	1.00 (.99–1.01)	.81		
Respiratory rate, breaths/min, mean (SD)	24.7 (6.6)	26.9 (7.9)	1.04 (.99–1.09)^b^	.11	^c^	
CSF WBC count, cells/µL^d^, median (IQR)	356 (64–1280)	215 (35–630)	0.49 (.37–.64)^b^	<.001	0.66 (.53–.80)	<.001
CSF culture positive for *Streptococcus pneumoniae*	160 (55)	165 (51.6)	1.06 (.66–17.1)^b^	.96	0.36 (.23–.57)	<.001
Plasma glucose, mmol/L, mean (SD)	7.5 (2.7)	7.6 (3.8)	1.00 (.95–1.05)	.87	^c^	
Hemoglobin, g/dL, mean (SD)	11.2 (2.6)	10.1 (2.6)	0.86 (.80–.91)^b^	<.001	0.84 (.78–.90)	<.001

Data are presented as No. (%) unless otherwise indicated.

Abbreviations: CI, confidence interval; CSF, cerebrospinal fluid; GCS, Glasgow Coma Scale; HIV, human immunodeficiency virus; IQR, interquartile range; MAP, mean arterial pressure; SD, standard deviation; SpO_2_, Oxygen Saturations (%); WBC, white blood cell.

a Performed on complete case data only (n = 523).

b Odds ratio for a unit change in predictor variable.

c Proportion of missing data >50%, so variable was excluded from multivariable analysis.

d Odds ratios calculated on log_10_ data. Fifteen patients were CSF culture negative with CSF WBC count >50 cells/µL; an additional 6 were HIV coinfected white cell counts >5 cells/µL.

The case fatality rate (CFR) was 44% (267/607) at day 10 and 54% (320/593) at day 40. The following associations with death at day 40 were observed univariately in the discovery cohort (Table 1): age, temperature, low Glasgow Coma Scale (GCS), low oxygen saturation, high pulse rate, low CSF WBC count, and low hemoglobin level (Table 1). Other included variables showed no association with outcome on univariate analysis: sex, HIV serostatus, mean arterial blood pressure, respiratory rate, CSF culture, seizures, and blood glucose (Table 1).

All variables tested were included in the multivariable analysis model, with the exception of oxygen saturation, temperature, and respiratory rate (>50% missing data) (Table 1). All variables with univariate significance, with the exception of age, also had an association with outcome on multivariable analysis (ORs per unit of measure change): GCS: OR, 0.77 [95% CI, .72–.83], *P* < .001; pulse rate: OR, 1.01 [95% CI, 1.00–1.03], *P* = .01; log_10_ CSF WBC count: OR, 0.66 [95% CI, .53–.80], *P* < .001; and hemoglobin: OR, 0.84 [95% CI, .78–.90], *P* < .001.

Although CSF pneumococcal culture had no significant association with outcome on univariate analysis, the multivariable model demonstrated a significant association with survival (OR, 0.36 [95% CI, .23–.57], *P* < .001). Outcome data by organism (Supplementary Table 1) showed that increased mortality was observed in patients with nonpneumococcal meningitis. Although numbers were small, increased mortality in the nonpneumococcal groups (CFR, 56.5% vs 51.5% for *S. pneumoniae*) was correlated with lower GCS and thus associated with poor outcome in the multivariable model. The lack of significant association with mortality on univariate analysis for the remainder of the variables, including HIV status, was retained on multivariable analysis.

### Results of the Nomogram

**Figure 2.**
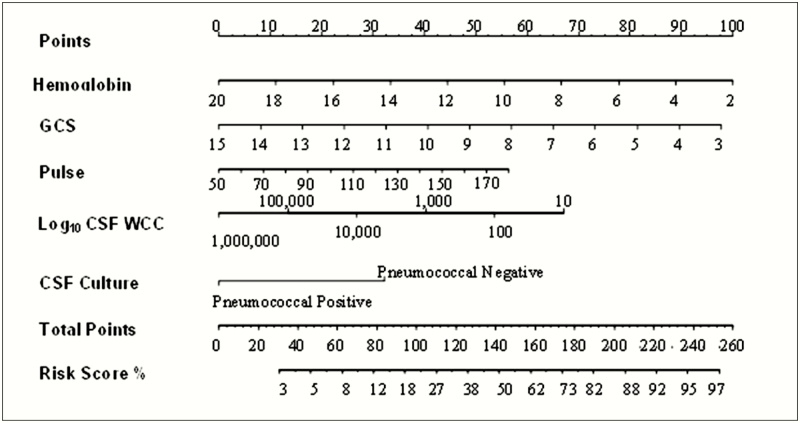
Malawi Adult Meningitis Score (MAMS) nomogram. A variable is measured and its value is matched to the corresponding point on the first row of the nomogram. The points are then added for all 5 variables for each patient. The total points for the patient are matched to the corresponding risk score percentage at the bottom (ie, match up the 2 last rows of the nomogram). Abbreviations: CSF, cerebrospinal fluid; GCS, Glasgow Coma Scale; WBC, white blood cell count.

Five variables strongly associated with day 40 mortality were used to develop the nomogram: GCS, pulse rate, CSF WBC count, pneumococcal culture, and hemoglobin level (Figure 2). To calibrate the nomogram, the continuous variables were categorized either by quartile around the median, or by standard normal clinical ranges. To optimize the nomogram, calibration data derived from the categorical data were compared with continuous data [30]. Categorical data led to overfitting and poorer performance; therefore, the nomogram using continuous data is reported (Figure 2).

The database was subject to 5000 bootstraps, and the entire derivation process was then repeated using the bootstrapped data. The log (OR) and associated bootstrapped CIs are reported (Supplementary Table 2). The data show shrinkage factor (bias) used to adjust the estimated regression coefficients in the final model for overfitting. Supplementary Table 2 details the bootstrapped CIs for estimated coefficients.

**Figure 3.**
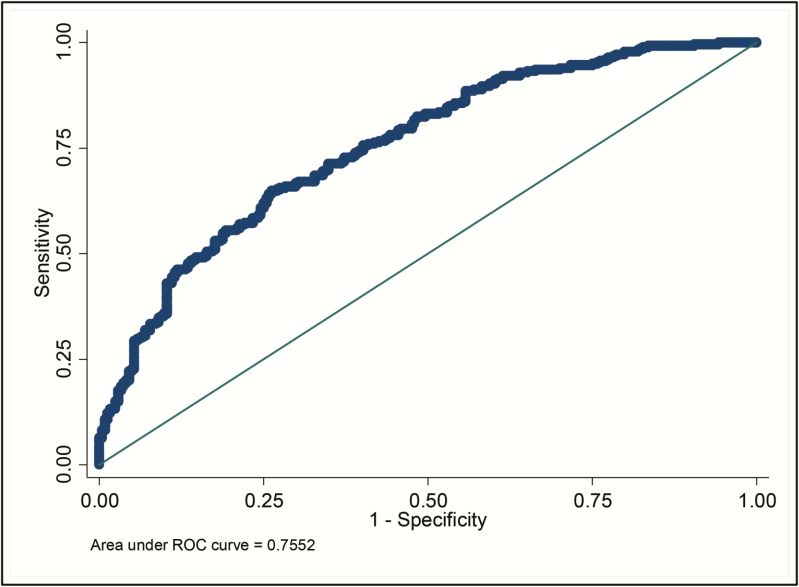
Receiver operating characteristic (ROC) curve for the Malawi Adult Meningitis Score (MAMS).

The corresponding ROC curve for the final nomogram is shown in Figure 3. The AUC was 0.76 (95% CI, .71–.80). The sensitivity of this model using a cutoff of 50% risk score is 71.7% (95% CI, 66.0%–76.9%) with a specificity of 63.1% (95% CI, 56.7%–69.2%).

### Calibration

Goodness-of-fit analysis was assessed to calibrate the nomogram. Hosmer-Lemeshow C-statistic was χ^2^ = 5.48, *df* = 8, *P* = .705. Calibration was good across individual centiles of estimated mortality probability (Table 2). The Brier score was 0.2 (95% bootstrap CI, .185–.215). Both the C-statistic and Brier score indicate good calibration. Discrimination was primarily determined by the C-index (AUC). The observed C-index was 0.755 from the original sample; the optimism corrected or adjusted C-index (mean over 5000 bootstraps) was 0.760 (95% bootstrapped CI, .71–.79).

**Table 2.** Summary of the Agreement of the Nomogram per Risk Centile

Group	Probability Centile of Death	Observed Deaths, No. (%)	Predicted Deaths, No. (%)	Total
1	0.234	6 (11.3)	9 (17.0)	53
2	0.326	14 (26.9)	15 (28.8)	52
3	0.389	23 (44.2)	19 (36.5)	52
4	0.460	24 (45.3)	22 (41.5)	53
5	0.540	25 (48.1)	26 (50.0)	52
6	0.604	30 (57.7)	30 (57.7)	52
7	0.677	30 (56.6)	34 (64.2)	53
8	0.763	40 (76.9)	37 (71.2)	52
9	0.836	42 (80.8)	42 (80.8)	52
10	0.962	45 (86.5)	46 (88.5)	52
Total		279	279	523

Hosmer-Lemeshow C-statistic, χ^2^ = 5.48, *df* = 8, *P* = .705.

MAMS, the European Meningitis Score (EMS), and an older outcome score developed in the United States were all derived using similar methods. Therefore, when the C-index of MAMS was compared directly to the published C-index of these scores [18, 19, 31], MAMS was equivalent. When tested using Malawi data, the EMS C-index was 0.68 (95% CI, .63–.73), compared with the MAMS C-index of 0.76 (95% CI, .71–.80), suggesting that MAMS may be a better score than EMS in this setting.

### Mortality Effects of Glycerol and Dexamethasone

Case fatality rate were compared by quartile of predicted risk in 2 clinical trials of dexamethasone and glycerol [9, 10]. In the dexamethasone study, an increased CFR was seen in the lowest risk quartile, but no differences were observed in other risk quartiles (Table 3). In contrast, glycerol was associated with increased risk of death across all but the highest risk quartile in that study (Table 3). The same trends were observed when nonbootstrapped data were used, but were not statistically significant (data not shown).

**Table 3.** Day 40 Mortality Outcomes From Dexamethasone and Glycerol Compared to Placebo by Malawi Adult Meningitis Score Risk Centile (Bootstrapped Data)

MAMS Risk Probability of Death at Day 40	Placebo (n = 1293)		Dexamethasone (n = 1268)		CFR% *P* Value
	Alive, No.	Dead, No. (CFR%)	Alive, No.	Dead, No. (CFR%)	
<25%	61	0 (0)	48	6 (11)	.010
25%–50%	232	106 (31)	236	126 (35)	.330
50%–75%	252	311 (55)	173	264 (60)	.100
>75%	66	265 (80)	99	316 (78)	.210
Total	611	682 (53)	556	712 (56)	.120

	Placebo (n = 549)		Glycerol (n = 592)		CFR% Significance of CFR Intervention vs Placebo *P* Value
	Alive, No.	Dead, No. (CFR%)	Alive, No.	Dead, No. (CFR%)	
<25%	51	6 (10)	49	14 (22)	.080
25%–50%	112	31 (21)	85	92 (52)	<.001
50%–75%	119	124 (51)	69	167 (70)	<.001
>75%	18	88 (83)	29	87 (75)	.140
Total	300	249 (45)	232	360 (61)	.001

Abbreviations: CFR, case fatality rate; MAMS, Malawi Adult Meningitis Score.

## DISCUSSION

This analysis shows that estimation of mortality risk from bacterial meningitis in adults in sub-Saharan Africa is possible using simple clinical parameters. MAMS is derived from community-acquired meningitis data, from an African region with a high HIV prevalence and burden of adult bacterial meningitis. This score is therefore likely to be relevant to similar settings but not to regions where epidemics of both *Neisseria meningitidis* and *S. pneumoniae* occur seasonally [2, 6, 32]. We demonstrate the utility of MAMS in the assessment of clinical trials, showing that compared to placebo, adjunctive glycerol is associated with the greatest increased risk of death in those patients predicted to have a lower risk of poor outcome. The only other score published from Africa was derived in the Sahel region and, therefore, while applicable to settings with epidemic of meningococcal meningitis [20], is of limited utility elsewhere.

We used similar data processes to develop MAMS as used in the EMS and a score developed in the United States [18, 19]. The EMS was developed on a discovery cohort of 691 patients [7] and underwent validation on 301 separate patients from the European Dexamethasone Study [33]. Patients receiving either dexamethasone or placebo were included and, interestingly, the authors comment that EMS performed better in those in receipt of dexamethasone who had better outcomes, compared with placebo [18, 33]. In the development of our score, we included patients who received dexamethasone, but excluded those receiving glycerol to minimize biases; glycerol was independently associated with mortality in Malawi [1, 9, 10]. The C-index of MAMS falls within the 95% CI for the C-index of both the EMS and the US-based score, and therefore can be considered of equal strength as the other scores in their validation settings.

The EMS performed less well in an external validation exercise, utilizing data from dexamethasone clinical trials from Vietnam and Malawi [9, 31, 34]. This may simply be due to differing methodology, including the use of categorized data in the EMS nomogram, and the selection of categorical cutoffs may have been less appropriate for these younger African patients [18]. However, we believe that the poorer performance of the EMS in those settings highlights critical differences between developed and developing country ABM populations, including longer time to presentation, a higher prevalence of HIV and anemia, greater frequency of complications, and fewer resources available for clinical management [35, 36].

The original trial of adjunctive glycerol in Malawi was stopped early due to unexpected increase in mortality in the glycerol arm [10]. Why this effect occurred predominately in those with lower predicted risk of death as determined by MAMS is uncertain. However, we hypothesize that in less severely ill patients, glycerol-induced vasodilation of pial microarterioles and osmotic effects may decompensate fragile but balanced fluid compartments in the brain and the systemic circulation, with adverse effects on brain swelling and perfusion [37, 38]. By comparison, in severely ill patients, breakdown of the blood–brain barrier is advanced; hence, glycerol has no additive adverse effect.

A major limitation in the development both of EMS and MAMS was missing data, managed using bootstrapping for both scores to reduce the risk of overfitting [18]. The developed model was validated to estimate the potential for overfitting and optimism in model performance using an internal validation procedure. This methodology is advised in the absence of external validation data, instead of use of split samples or cross-validation without replication [25, 26]. The nomogram was optimized using standard validation exercises to reduce overlap, and high levels of agreement were observed. Cohort assembly was done using data from multiple studies in the same hospital; biases from interobserver disagreement were minimized by only selecting variables that were collected uniformly across all studies. All data were collected prospectively in the same units, by research staff trained in the same institution, using the same equipment to minimize potential biases associated with post hoc cohort assembly. We used data from the first recording after admission to hospital in all studies to minimize the effect of in-hospital delays on alterations on physiological variables that may otherwise affect the prognostic power of the score. Other limitations include use of data from only 1 country, relative infrequency of HIV-uninfected patients, lack of inclusion data on morbidity endpoints and processes of care including antibiotic delays, and relatively few patients with meningococcal vs pneumococcal meningitis [6]. The MAMS nomogram requires external prospective validation in other populations, including specific validation in countries at risk of epidemic meningococcal disease.

It is clear that in the development of a severity score, other factors beyond those measured in the score may be important in determining outcome of adults with bacterial meningitis in sub-Saharan Africa. Although HIV coinfection was not associated with mortality and not included in the nomogram, it is possible that low CSF WBC count and low hemoglobin level may both correlate with advanced immunosuppression. CD4 cell count data are currently lacking in this setting.

In conclusion, the MAMS risk stratification tool has a good predictive power for use in ABM in adults in sub-Saharan Africa outside the epidemic meningitis belt. MAMS can be used in the analysis of prospective clinical trial data, testing efficacy of interventions in different risk groups. The differences between MAMS and EMS scores highlight important differences between ABM patients in these contrasting environments. Further work is required to explore these differences, to test MAMS prospectively, and to design optimal interventions to reduce the unacceptable mortality from meningitis in Africa.

## Supplementary Material

Supplementary Data

## Notes

***Acknowledgments.*** The authors thank Philip Gichiru for his help with the generation of the original Malawi database; Malango Msukwa for his assistance with data from the Bundles of care for Adult Meningitis trial; and Professor Diedrik van de Beek for his sharing his experience with the generation of meningitis severity scores. In addition, we thank Professor E. Ziljstra for his support of the data collection for both the steroid and glycerol trials.

***Disclaimer.*** The funding body had no role in study design, data collection, data analysis, data interpretation, or writing of the report. The corresponding author had full access to all the data in the study and had final responsibility for the decision to submit for publication.

***Financial support.*** This work was supported by a clinical PhD fellowship in Global Health (089671/B/09/Z) to E. C. W. from the Wellcome Trust; and the MLW core grant from the Wellcome Trust. The original constituent studies in the discovery cohort were funded by the Meningitis Research Foundation (Steroids for Adult Meningitis and Glycerol for Adult Meningitis) and by the MLW core grant from the Wellcome Trust.

***Potential conflicts of interest.*** Author certifies no potential conflicts of interest. All authors have submitted the ICMJE Form for Disclosure of Potential Conflicts of Interest. Conflicts that the editors consider relevant to the content of the manuscript have been disclosed.
