# Supplementary material for: Prediction of Outcome From Adult Bacterial Meningitis in a High-HIV-Seroprevalence, Resource-Poor Setting Using the Malawi Adult Meningitis Score (MAMS)
Source: Clin Infect Dis. 2016 Dec 2;64(4):413–9. doi: 10.1093/cid/ciw779 (PMC5399948; doi:10.1093/cid/ciw779)
Supplement: Supplementary Data [file ciw779_Supplementary_Data.zip › MAMS_supplementary_table_for_CID_15_10_16_Final.docx]

**Prediction of outcome from adult bacterial meningitis in a high HIV seroprevelence, resource-poor setting using a new severity scoring tool, the Malawi Adult Meningitis Score (MAMS).**

**Supplementary Tables**

**S.table 1 CSF culture rates for all meningitis patients in the meningitis database, used in the univariate analyses by outcome group n=593**

| Organism | Outcome at day 40 |  |  |
| --- | --- | --- | --- |
|  | Alive  n=273 | Dead n=320 | CFR |
| Culture negative | 93 | 125 | 0.57 |
| *Neisseria meningitidis* | 15 | 1 | 0.06 |
| *Haemophilus influenzae* | 3 | 0 | 0 |
| Non-meningococcal gram negative organisms* | 2 | 18 | 0.90 |
| *Streptococcus pneumoniae* | 155 | 165 | 0.51 |
| Other organisms† | 4 | 10 | 0.71 |

*= predominately E.coli and non-typhoidal *S*almonellae. † Other organisms including *S.aureus*, group A Streptococci and non-pneumococcal alpha haemolytic streptococci.

These data include all patients in the Malawi meningitis database used for the univariate analysis in the derivation of MAMS. A sub-set of complete case data were used in the multivariate analyses n=523.

**S.Table 2 Results of Multivariable logistic regression of day 40 outcome on covariates, boostrapped data.**

| Covariate | OR (95%CI) | Bias | Significance (p) |
| --- | --- | --- | --- |
| CSF White cell count (cells/mm^3^)** (log_10_) | 0.66 (0.52 : 0.81) | 0.99 | <0.001 |
| Haemoglobin (g/dL) | 0.84 (0.78 : 0.90) | 1.00 | <0.001 |
| Pulse (beats/minute) | 1.01 (1.00 : 1.03) | 1.00 | 0.017 |
| Glasgow Coma Score | 0.77 (0.72 : 0.83) | 1.00 | <0.001 |
| CSF culture positive for  *S. pneumoniae* | 0.36 (0.23 : 0.58) | 0.98 | <0.001 |
| *constant* | *202.96 (28.7 : 1472.9)* | *1.12* | *<0.001* |
